# Strengthening Rare Disease Data Readiness: a semantic approach to cross-registry interoperability

**DOI:** 10.1038/s41597-026-08406-6

**Published:** 2026-09-30

**Authors:** Clemens M. Hentrich, Jessica Vasseur, Ines Reinecke, Jens Weidner, Jens Göbel, Holger Storf, Annette Grüters-Kieslich, Franziska Krause, Mara Hartung, Martin Sedlmayr, Michele Zoch

**Affiliations:** 1 Institute for Medical Informatics and Biometry, Faculty of Medicine and University Hospital Carl Gustav Carus, TUD Dresden University of Technology, Dresden, Germany; 2 Goethe University Frankfurt, University Medicine, Institute of Medical Informatics (IMI), Frankfurt am Main, Germany; 3 Data Integration Centre, Faculty of Medicine and University Hospital Carl Gustav Carus, TUD Dresden University of Technology, Dresden, Germany; 4 Institute for Experimental Paediatric Endocrinology, Charité – University Medicine Berlin, Berlin, Germany; 5 Eva Luise und Horst Köhler Stiftung für Menschen mit Seltenen Erkrankungen, Bonn, Germany; 6 Business Division Strategic Science Management, Berlin Institute of Health (BIH) at Charité – University Medicine Berlin, Berlin, Germany

## Abstract

Rare diseases affect an estimated 446 million people worldwide, yet reliable epidemiological data are scarce due to insufficient data sources and lack of standardization. The German National Registry for Rare Diseases (NARSE) aims to establish a comprehensive epidemiological data basis and enable data availability for research. However, limited interoperability between clinical and epidemiological data sources restricts secondary use and cross-registry research. This study assesses the semantic interoperability between NARSE and two core data sets: the German Medical Informatics Initiative (MII) core data set and the European Rare Disease Registry Infrastructure (ERDRI) core data set. Using a formal consensus process, mapping categories (exactMatch, closeMatch, broadMatch, narrowMatch, relatedMatch) were defined, analyzed, and evaluated for information loss using a heuristic scale. Most NARSE elements aligned with the MII core data set, while mapping to the ERDRI core data set revealed more challenges due to undefined value sets and inconsistent coding systems. These findings highlight the importance of consistent application of coding standards to safeguard information value and improve interoperability in rare disease research.

## Introduction

It is estimated that 446 million people worldwide are affected by rare diseases, which, in the European Union (EU), affect fewer than 5 in 10,000 individuals^1^. While there is no universally accepted definition of rare diseases, most national definitions are based on prevalence. However, these prevalence figures are not derived from evidence-based findings due to insufficient epidemiological data and a variety of disparate information sources^2^.

Reliable data, such as that collected through patient registries, is essential to address this gap. The German National Registry for Rare Diseases (NARSE) was established as Germany’s first cross-disease registry to build a comprehensive epidemiological foundation for rare diseases^3,4^. Its primary goal is to generate high-quality data to establish a comprehensive epidemiological data basis, while its secondary objective is to enable high-quality data for national and international research.

For registry data to fulfill this potential, it must achieve data readiness, defined as the state where all four pillars are sufficiently met to enable data use. The Data Readiness framework^5^ conceptualizes how semantic and syntactic interoperability, data quality, data availability and data provenance could collectively enable cross-registry research. Data readiness is not merely about accessibility but about preserving meaning and context during data exchange.

Data readiness is the overarching prerequisite for enabling secondary research, large-scale analyses, and cross-registry insights, particularly in rare diseases where patient cohorts are small and fragmented^6^. This requires semantic interoperability: the ability to integrate and interpret data from heterogeneous sources without ambiguity or loss of meaning^7^. Semantic interoperability depends on alignment with standardized frameworks, such as the German Medical Informatics Initiative (MII)^8^ and European Rare Disease Registry Infrastructure (ERDRI)^9^, and is closely linked to data quality (e.g., completeness or “fitness for purpose”) and data provenance (traceability of data lineage)^5,10^.

Despite growing emphasis on data readiness, a systematic semantic mapping between NARSE and established core data sets (CDS), namely the MII CDS and ERDRI CDS, has not been implemented. This gap impedes semantic interoperability, forcing manual data re-entry across systems, increasing documentation burdens, and risking inconsistencies. Without such mapping, national registry data cannot be seamlessly translated into formats suitable for international research infrastructures, such as the European Reference Networks (ERNs) building on ERDRI CDS, nor reliably integrated with local Data Integration Centres (DIC)^11^ and routine care data sets.

This study addresses this critical barrier by developing a semantic mapping between NARSE and the different CDS in order to determine whether cross-registry data flows are semantically feasible. Building on this primary aim, the mapping is designed to achieve three operational goals for data readiness: (1) Minimize semantic information loss during data transfer from local systems to national registries and international reuse contexts, (2) reduce redundant data capture by enabling reuse of standardized data elements, and (3) align data structures to provide a sufficient basis for harmonized, cross-registry analyses.

This manuscript does not present the operational goals as its primary deliverables. Instead, it assesses the extent to which the implemented semantic mapping realizes those objectives and quantifies the residual information loss. By enabling automated, interoperable data flows, this work directly strengthens data readiness for rare disease research. In the long term, it supports cross-registry analyses, enhances care quality, and advances the European Health Data Space (EHDS) vision^12^.

## Materials and Methods

### NARSE data set

The NARSE data set was designed by the Think Tank of the Eva Luise and Horst Köhler Foundation for People with Rare Diseases (ELHKS), which convenes interdisciplinary experts in medicine, research, policy, and data science^13^. This initiative specifically addressed gaps in existing rare disease standards by building upon the ERDRI Common Data Set (CDS) while incorporating critical additions such as brief family history for rare diseases.

The current NARSE data set (Version 1.2 as of 30 January 2025)^14^ comprises a total of 43 data elements from different categories of data with distinct storage locations and safeguards – identifying data, consent data, and medical data. The data elements comprising the medical data set are represented in a tabular format reflecting the structure of the electronic case report forms (eCRFs). The data set integrates internationally recognized semantic standards to ensure interoperability: diagnostic information uses ORPHAcodes and versioned ICD codes, clinical phenotypes are documented via the Human Phenotype Ontology (HPO). At the same time, the data set also contains registry-specific data elements, such as “Inclusion date” and several elements related to informed consent. Due to their registry-specific nature, predefined exclusion criteria were applied, specifying that data elements exclusively relevant to a particular registry were not considered for the mapping. Consequently, 11 data elements of the NARSE data set were excluded, leaving 32 data elements for the semantic mapping. A complete list of the excluded elements is provided in Table 1.

**Table 1:** Data elements were excluded from the mapping based on the predefined exclusion criteria.

Category	Elements	Reason for exclusion
Informed consent	Consent date; Consent for data use; Data use international (EU); Capture of genetic information; Case review; Contact for study or therapy information; Contact for networking	Informed consent procedures are registry-specific and therefore not suitable for cross-registry comparison
Diagnosis	Inclusion Date	Registry-specific administrative information
Personal information	Age group at inclusion	Registry-specific administrative information
Identifying data	Phone; E-Mail	Identifying information collected solely for participant re-contact and therefore registry-specific

All data elements are publicly accessible through two resources: (1) A complete overview of the current NARSE data set is provided as a tabular data dictionary^14^, including specifications of data formats and indications of whether a field is mandatory or optional (2) a formal metadata repository^15^ based on Samply.MDR^16^ implementing the ISO 11179 standard for metadata.

### Reference data sets

The mapping process utilized two reference data standards: the MII CDS and the ERDRI CDS. These were selected for their complementary technical approaches to rare disease data standardization.

MII CDS is specified through the international communication standard Health Level 7 (HL7) Fast Healthcare Interoperability Resources (FHIR)^17^ profiles, supported by Unified Modeling Language (UML) diagrams and detailed implementation guides^18,19^. This formalized structure enables robust technical interoperability. This is particularly relevant in the federated architecture of the MII, where the CDS is implemented locally across different modules at each site. The MII follows a decentralized data-storage paradigm and the CDS plays a central role in maintaining structural and semantic consistency across these implementations^20,21^. The mapping was performed using the publicly available MII CDS implementation guides, including the modules Diagnosis (v2025.0.0), Person/Patient (v2025.0.0), Molecular Genetics (v2025.0.0 DE), Symptoms (v2024.0.0-ballot), Laboratory Findings (v2025.0.2 DE), and Procedures (v2025.0.0).

ERDRI CDS is documented in tabular form and includes recommendations for preferred terminologies for rare disease research^22^. As no versioned releases of the ERDRI CDS are available, the published specification referenced here was used for the mapping. It comprises only 16 data elements, intentionally designed as a minimal applicable core for all rare diseases. Of these, six relate to administrative processes, research consent or biobanking rather than clinical content. These elements were therefore excluded from the semantic mapping, consistent with the exclusion criteria applied to the NARSE data set (Table 1). Of the remaining ten clinically relevant elements, four are represented in the underlying data models in both categorical and date-based forms (age at onset, age at diagnosis, diagnosis coding, and genetic diagnosis). Thus, 14 elements were assessed in the semantic mapping. Its concise structure prevents limitations arising from complexity while allowing disease-specific extensions where needed^6,23^. While less formally structured than the MII CDS, it provides practical guidance for clinical data collection in rare disease contexts.

### Methodological approach

An interdisciplinary team of three researchers with expertise in rare disease data standards developed the mappings between NARSE and the ERDRI or MII CDS using a formal consensus process^24^. All team members were thoroughly familiar with the NARSE data set and reference CDS specifications. The mapping process followed a structured three-phase workflow: (1) Initial mapping: an initial draft of the mapping was provided, to which the researchers suggested and incorporated improvements (2) Iterative refinement: the resulting mappings were categorized independently by each researcher using SKOS-based relations^25^ (Table 2) (3) consensus building: discrepancies were resolved through team discussion. Unanimous agreement was targeted for all mappings, unresolved cases were resolved by a 2/3 majority vote.

**Table 2:** Mapping categories, drawn from SKOS vocabularies^25^.

Category	Description	Preservation score		Example
exactMatch	Both sides are identical and the contents are transitive	Lossless	1.0	ORPHAcode: ORPHAcode
closeMatch	Both sides are similar, but not identical and not transitive	Low-loss	0.75	Patient gender: Administrative gender
broadMatch	One side is broader (more general) than the other	High-loss	0.5	Not observed in this study
narrowMatch	One side is narrower (more specific) than the other	High-loss	0.5	Not observed in this study
relatedMatch	Both sides are associatively related.	High-loss	0.5	Condition: Affected family members: rare disease

While SKOS relations are traditionally applied to map discrete, 1:1 schema elements (element-level mapping), our approach shifted toward content-level correspondence to accommodate structural differences between the data models. For instance, a classical element-level mapping would fail when aligning a single composite registry field in NARSE, such as “family history,” with the highly modular MII CDS, which represents family history across nested HL7 FHIR resources (e.g., FamilyMemberHistory containing multiple sub-elements). In such cases, we mapped the overall semantic content (the capacity to record familial genetic risks) rather than forcing an artificial schema-to-schema element alignment. This content-level focus allows us to evaluate whether the clinical intent of the data is preserved during transfer, though it limits direct technical comparability with classical, strict vocabulary-matching studies.

To quantify the mapping, preservation scores were assigned per category (Table 2). The overall preservation score was calculated as:

$$P=\sum _{i}\frac{{c}_{i}}{n}\cdot {w}_{i}$$

where ${c}_{i}$ is the number of elements assigned to SKOS match category i, ${w}_{i}$ is the corresponding preservation weight (Table 3), and n is the total number of elements assessed for the respective comparison. Elements for which no corresponding element could be identified in the reference data set (“no_mapping”) are included in n but do not contribute a term to the sum and are reported separately to indicate overall mapping coverage. Information loss rate was defined as 1 minus preservation score.

**Table 3:** Alternative weighting schemes used in the sensitivity analysis of the preservation score.

Category	Primary	Conservative	Equidistant	Liberal
exactMatch	1.0	1.0	1.0	1.0
closeMatch	0.75	0.75	0.8	0.85
broadMatch	0.5	0.6	0.6	0.65
narrowMatch	0.5	0.4	0.4	0.65
relatedMatch	0.5	0.2	0.2	0.45

The assigned weights should not be interpreted as a quantitative representation of the formal SKOS semantics but as heuristic values intended to approximate the relative degree of semantic information preservation during data transfer. The normalized heuristic scale is conceptually aligned with established metrics used for mapping evaluation^26^ and provides a simple and transparent way to quantify potential information loss in mappings. It is explicitly defined rather than statistically derived, offering a practical framework to compare mapping outcomes and evaluate data availability without unnecessary complexity. As a sensitivity analysis, the preservation score was calculated using four weighting schemes for the SKOS match categories (Table 3). The weighting schemes ranged from a more conservative to a more liberal interpretation of closeMatch and relatedMatch.

### Technical implementation

**Fig. 1:**
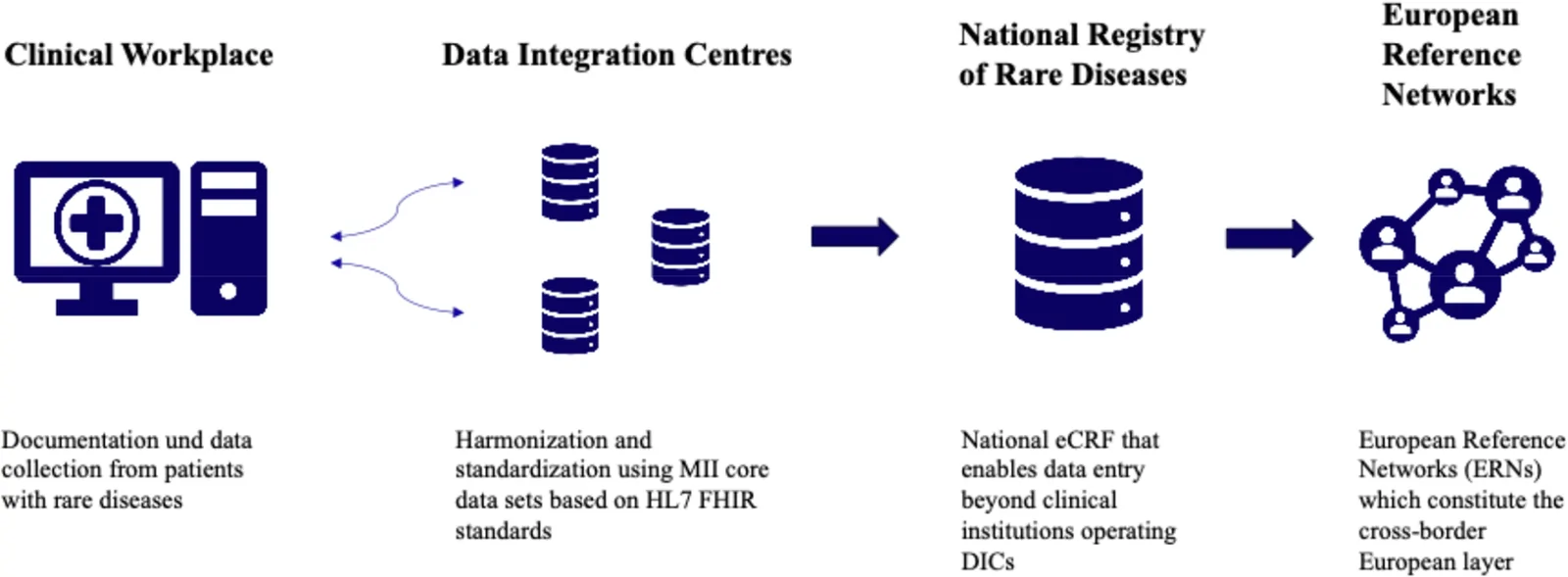
Data flow from the MII CDS (source) into NARSE (target), and from NARSE (source) into the ERDRI CDS (target).

As the mapping follows a potential data flow for a data transfer (Fig. 1), the results table incorporates this directional aspect. For the MII to NARSE comparison, the source is the MII CDS and the target is NARSE. Accordingly, for each of the 32 in-scope NARSE target elements, we assessed whether a corresponding MII CDS source element exists that could populate it, reflecting the transfer of clinical routine data into the registry. For the NARSE to ERDRI comparison, the source is NARSE and the target is the ERDRI CDS. We assessed for each of the 14 included ERDRI CDS target elements whether a corresponding NARSE element exists that could populate it, reflecting the transfer of registry data to the European infrastructure. The results are publicly available^27^ and stored within a Transition Database (TransDB) to facilitate harmonization with other mappings^28^. It provides an implementation-neutral design, capable of mapping to FHIR resources, conventional tabular formats, and MDR schemas, providing a human-readable format.

### Ethics statement

Ethics approval and consent to participate not applicable because this study was based exclusively on publicly available published specifications and did not involve the analysis of patient-level or other human-subject data.

## Results

### Mapping

Table 4 provides a brief overview of the mapping by target data set, how coverage is distributed across the individual categories. These were defined based on the structural organization of the NARSE eCRFs and do not represent standardized classifications. Individual results on the data element level are publicly available^27^ and can be accessed through the TransDB. The complete element-level mapping results are provided in Supplementary Table S1.

**Table 4:** Coverage of data categories in the German National Registry for Rare Diseases (NARSE), German Medical Informatics Initiative (MII) core data set (CDS) and European Rare Disease Registry Infrastructure (ERDRI) CDS ( = available,  = limited availability,  = no answer possible (e.g. due to missing data)).

Category	Data set		
	NARSE	MII CDS	ERDRI CDS
Diagnosis			
Personal information			
Family history			
Anamnesis			
Identifying data			

Specifically, for 30 of the 32 NARSE target elements, a corresponding element in the MII CDS was identified, indicating that these NARSE fields could be populated from MII data. Among the mappings, 31% were classified as “exactMatch”, 53% as “closeMatch” and 9% as “relatedMatch”. Notably, no mappings fell into the “broadMatch” or “narrowMatch” categories. The only elements that could not be mapped (“no_mapping”) were “biological sex at birth” and “place of birth”. Of the 32 data elements, five required a consensus discussion due to disagreement between the initial reviewers. For three elements, consensus was reached on “closeMatch”, although “exactMatch” was also considered. For the remaining two elements, the decision was between “relatedMatch” and “no_mapping”, with both ultimately being classified as “relatedMatch”.

With regard to the alignment between NARSE and ERDRI CDS, a total of 14 elements were assessed, of which 13 could be semantically mapped to corresponding elements in the ERDRI CDS. Of these, 21% were classified as “exactMatch”, 64% as “closeMatch” and 7% as “relatedMatch”. One data element could not be mapped (“no_mapping”). This indicates that the essential components (e.g. diagnosis) of the ERDRI CDS are well represented within NARSE. However, an exception is the International Classification of Functioning, Disability and Health (ICF)^29^, which is not included in the NARSE. Of the 14 data elements assessed against the ERDRI CDS, three required a similar consensus discussion. All three were free-text elements and, following discussion, were assigned to the closeMatch category.

The results show that data integration across the relevant data flows is not entirely lossless but can still be considered low-loss. In general, the proportion of “high-loss” mappings is very small, both in the first step from the MII CDS to NARSE and in the second step from NARSE to ERDRI CDS.

To assess the robustness of the overall preservation score, the analysis was repeated using alternative weighting schemes (see Table 3 and Methods). Across all weighting schemes, the preservation score ranged from 0.73 to 0.81 for the MII CDS and from 0.71 to 0.79 for the ERDRI CDS (Table 5). The relatively small variation across the tested weighting schemes indicates that the preservation score is robust to the choice of category weights.

**Table 5:** Preservation scores calculated according to the weighted preservation score described in the Methods section.

Category	Data set		
	Scale	MII to NARSE (n = 32)	NARSE to ERDRI (n = 14)
Lossless	1	0.31	0.21
Low-loss	0.75	0.40	0.48
High-loss	0.5	0.05	0.04
**Overall preservation score (Primary)**	[0,1]	**0.76**	**0.73**
*Sensitivity analysis (alternative schemes, see* Table 3*, Methods)*			
Conservative	[0,1]	0.73	0.71
Equidistant	[0,1]	0.76	0.74
Liberal	[0,1]	0.81	0.79

The alignment from MII CDS to NARSE assessed the representation of the 32 NARSE elements and achieved an overall preservation score of 0.76 (24% information loss). The alignment from NARSE to ERDRI assessed the representation of the 14 ERDRI CDS elements and achieved a preservation score of 0.73 (27% information loss). Because the two scores are computed over different reference sets, they quantify conceptually distinct questions and are not directly comparable in magnitude. Both mappings nonetheless indicate that the observed information losses were predominantly minor, with most non-exact mappings falling within the low-loss category.

**Fig. 2:**
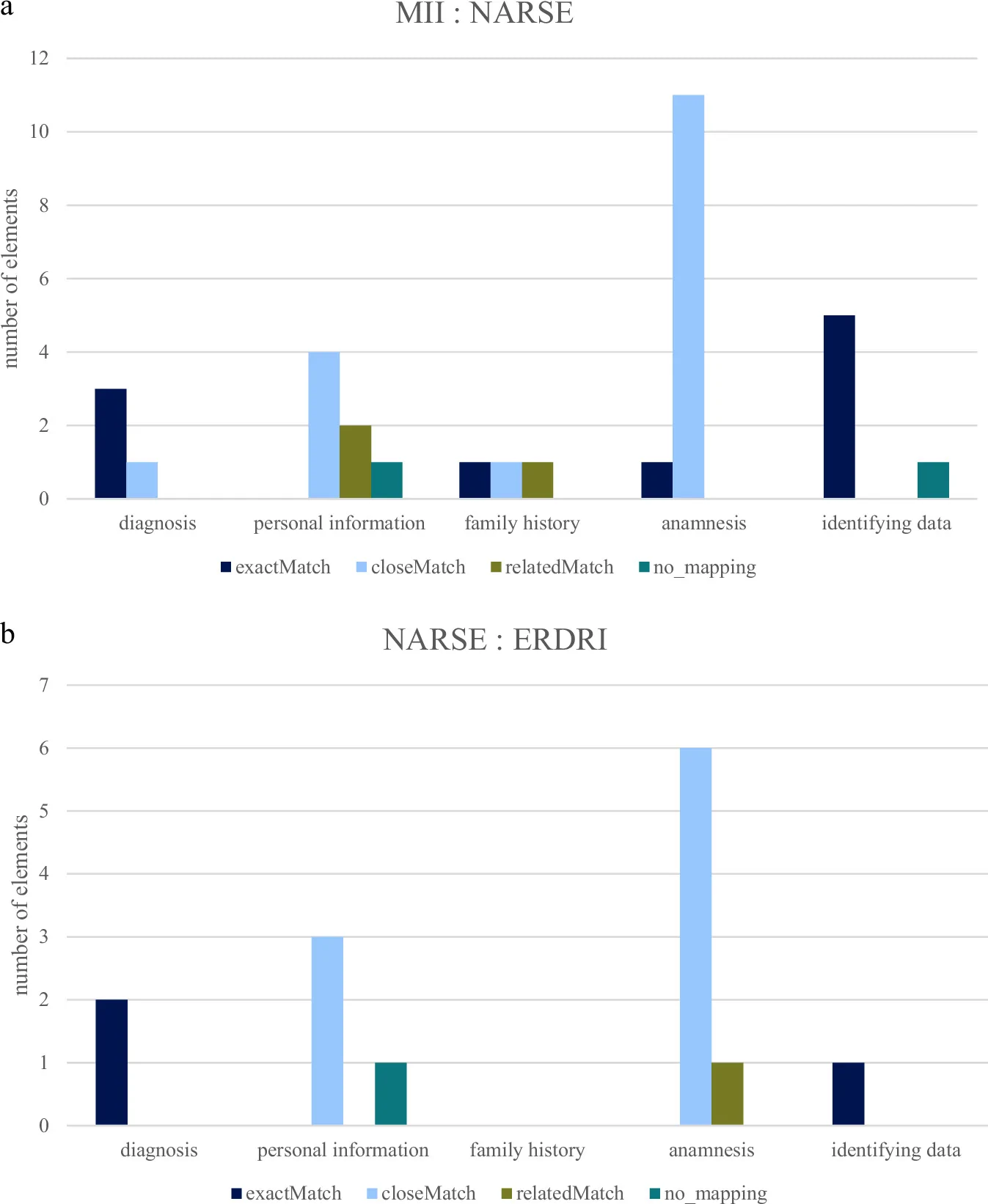
(**a**) Elements mapped per data category. German Medical Informatics Initiative (MII) core data set (CDS) to German National Registry for Rare Diseases (NARSE). (**b**) Elements mapped per data category. German National Registry for Rare Diseases (NARSE) to European Rare Disease Registry Infrastructure (ERDRI).

Figure 2 visualizes the quantitative mapping results stratified by the data categories used in Table 4. Based on these results, preservation scores were derived for each category. While the previous analysis focused on the data availability, these results reflect the quality of the semantic mapping within each thematic category.

For the alignment between MII CDS and NARSE the highest preservation scores were observed in the categories diagnostics (0.94) and identifying data (0.83). The remaining categories showed scores ranging between 0.57 and 0.77.

In the alignment from NARSE to ERDRI the preservation score for diagnostics reached 1.0, while other categories showed lower scores down to 0.56. No preservation scores could be determined for the categories family history due to missing corresponding elements.

## Discussion

### Context and purpose

The compatibility of NARSE with the MII CDS and ERDRI CDS is of central importance for rare disease research in Germany. The results of this study show that, despite their different objectives and designs, the data models provide a good basis for semantic alignment and interoperability.

The differences between the data sets can largely be attributed to their differing context, purpose, and level of technical implementation. NARSE serves as a national registry for rare diseases in Germany, with a primary focus on collecting epidemiological data. At this stage, data is captured by manual data entry in eCRFs, and the NARSE data model is designed to reflect a harmonized yet flexible framework for a broad range of rare diseases. In contrast, the MII CDS is a standardized data and information model to enable interoperable secondary use of clinical routine data across participating university hospitals via Data Integration Centres. It has a modular structure in which not all modules are mandatory, and semantics are more tightly integrated with operational healthcare data models, focusing on longitudinal clinical data. The ERDRI CDS provides a harmonized set of core data elements to support interoperability across different ERN registries. While also focused on epidemiological data, the ERDRI CDS is designed as a foundational model that can be extended domain-specifically^30^.

Given the structural and semantic discrepancies between the data sets, further alignment may be beneficial. The mapping to the MII CDS was complicated by the fact that information can appear in different modules, allowing for interpretational variability across different locations. Therefore, attention is drawn to efforts to ballot an MII CDS extension module for rare diseases. This module enables the targeted integration of routine data in a single location, thereby reducing the scope for interpretation across sites.

### Discussion of the methodology

The mapping was successfully conducted using a formal (nominal) consensus process, which enabled a structured evaluation of the mapping quality using a normalized heuristic scale. A formal inter-rater reliability measure such as Fleiss’ κ could not be calculated, as individual categorizations prior to consensus were not retained. This limitation is also relevant to comparable SKOS-based mapping studies that rely on consensus-based workflows. However, a post-hoc review of the discussion records suggests that disagreements occurred mainly in the closeMatch category, indicating that ambiguity was not uniformly distributed across match types. Future harmonization efforts should prospectively document individual pre-consensus judgments to enable formal reliability assessment.

While the overall approach proved effective for establishing semantic alignment, several methodological considerations should be acknowledged. Due to the generic structure of the concepts precise one-to-one mapping was not always feasible. As described in the Methods section, our shift from strict element-level to content-level correspondence means that the mapping does not fully conform to a strict element-level application of the SKOS mapping model. While this was necessary to bridge the structural gap between flat registry tables and hierarchical FHIR profiles, it introduces a level of qualitative interpretation by the mapping experts. Consequently, the reliance on manual consensus remains a potential source of bias that future, more automated mapping tools should aim to minimize.

The classification of elements as “high-loss” does not necessarily reflect practical information loss in real-world applications. For example, elements categorized as “relatedMatch” may retain their information in one mapping direction but result in information degradation in the reverse direction. Furthermore, the handling of complete data loss, represented by a theoretical score of 0, remains unresolved. In practice missing mappings do not necessarily equate to a total absence of information. The data can still be manually entered into registry systems. Thus, the reported information loss primarily reflects limitations for automated data transfer. It is especially relevant under the assumption that information loss may accumulate along the data flow. Even small, quantitatively low-losses in the upstream source may translate into substantial real losses in the target system.

Another important consideration is that the current methodology does not account for whether a data element is mandatory or of secondary importance within the target system. Consequently, the calculated information loss rate may overestimate the practical impact on data quality and data availability.

Future work should aim to refine the evaluation metrics by incorporating field-level metadata such as the criticality of data elements and the directionality of mappings. This also includes applying element-level weighting in the calculation of information loss rates, so that fields of particular clinical importance (e.g. ORPHAcodes) are given higher impact within the heuristic scale. In addition, strategies to reduce reliance on manual consensus (e.g. semi-automated or ontology-driven mapping tools) may help minimize potential bias.

### Interpretation of the results

The results demonstrate that the data sets share a largely comparable semantic structure across categories, thereby providing a solid foundation for semantic alignment and interoperability. The majority of NARSE elements could be successfully mapped to corresponding elements in the MII CDS and essential components of the ERDRI CDS were also represented within NARSE. This structural comparability is encouraging as it supports the feasibility of establishing harmonized data flows between national and European infrastructures for rare diseases to increase data availability.

It should be noted that a mapping is only meaningful if the data elements are present in the target data set. For each mapping, the preservation score is calculated with the target data set as the reference. Thus, for the MII-to-NARSE mapping, we assess whether each of the 32 in-scope NARSE elements can be populated from the MII CDS. For the NARSE-to-ERDRI mapping, we instead assess whether the clinically relevant ERDRI elements can be populated from NARSE. This is particularly important for ERDRI because its CDS is deliberately designed as a minimal core data set. Considering all NARSE elements in the ERDRI score would therefore result in many “no_mapping” entries that reflect the limited scope of ERDRI rather than information loss. The two preservation scores therefore reflect different reference directions and should not be interpreted as directly comparable measures of overall information loss.

The mapping reveals that a high proportion of elements were classified as “exactMatch” or “closeMatch”. This suggests strong semantic proximity between the investigated data sets. The absence of the “broadMatch” or “narrowMatch” categories indicates that the mappings are generally precise rather than loosely related. Nevertheless, the comparatively smaller proportion of “exactMatch” mappings between NARSE and ERDRI CDS points to challenges in data harmonization, particularly when different registry frameworks follow diverging design principles^31^.

A more critical view emerges when examining the individual data categories based on the structure of the NARSE data set, particularly anamnesis, diagnostics and personal information. For personal information, most discrepancies arise from differences in representing age and sex/gender, with the lack of a clear distinction between the latter two posing potential limitations. Only NARSE captures both dimensions, which could become highly relevant in contexts such as therapy recruitment. In Anamnesis, the observed information loss is largely attributable to the divergent use of value sets.

Overall, these findings underscore that the highest rate of information loss are linked to insufficient use of standards. Conversely, the consistent application of established coding systems, such as ORPHAcodes or ICD codes, effectively safeguards information value and ensures greater interoperability.

The ERDRI CDS offers a limited representation of the patient’s personal and familial background. Since over 75% of rare diseases have a genetic origin^32^, the consideration of the family history was an important aspect in the development of the NARSE data set. Detailed information on family history can be crucial for understanding inheritance patterns, distinguishing the risk of individuals and enabling genetic counseling. The lack of the disability classification according to the ICF beyond the ERDRI CDS represents a notable gap in NARSE that should be addressed in future harmonization efforts.

### Automation of data transfers

Data quality is essential in rare disease research, where data are often scarce and heterogeneous^33^. Standardized and interoperable data are critical for meaningful analyses. The consistent use of terminologies and standards, such as ORPHAcodes, minimizes reliance on free-text fields and enhances data integrity. However, the theoretical nature of the mapping process presented here highlights the need for further infrastructural and organizational developments. A round-trip test at the patient level, as described in previous studies^34^, is necessary to evaluate data preservation during mapping processes, though it requires additional preparatory steps and informed consent.

The automation of data transfers and ensuring high data quality are critical for advancing rare disease research, yet they face significant challenges. Legal and regulatory frameworks, particularly the General Data Protection Regulation (GDPR), impose strict limitations on data sharing, even within the European Union. Informed consent, data privacy, and security are paramount, and broad consent models, such as those used by the MII^35,36^, are insufficient for this purpose. Each participating institution must establish mechanisms to verify the legality of data transfers.

Despite these efforts, centralized data storage poses significant challenges, particularly for rare diseases. Theoretical information loss rates, existing secure data infrastructures at individual sites, and the heightened risk of patient re-identification in rare diseases make centralized storage less beneficial. Rare diseases often involve small patient populations, increasing the risk of re-identification, especially with detailed clinical and genetic data.

In this context, the mapping process serves as a critical step toward enabling distributed analyses. By harmonizing data structures and ensuring semantic interoperability, it facilitates data sharing across registries while preserving patient privacy. This approach aligns with legal and ethical constraints, minimizing the need for centralized storage and reducing re-identification risks. Ultimately, it supports the vision of cross-registry interoperability, where harmonized data sets can be analyzed in a distributed manner, thereby strengthening rare disease data readiness and advancing research.

## Conclusions

This work highlights the relevance of incorporating semantic standards directly at the point of data capture in clinical registries. Establishing semantic interoperability upstream, during initial documentation, reduces the need for retrospective standardization, minimizes information loss, and supporting more automated data flows across heterogeneous systems. By aligning data collection with FAIR principles from inception, this approach has the potential to strengthen national infrastructures and prepare rare disease data for European frameworks like the EHDS.

Future work needs to investigate how semantically enriched data entry interfaces can support structured and user-friendly clinical documentation and secondary use. Such interfaces are expected to improve data quality, reduce redundant data entry, and ensure that collected data is inherently interoperable and analysis-ready.

The results underline the central importance of semantic interoperability as a prerequisite for data availability in national and cross-border research. The alignment with established standards fosters the sustainable integration of rare disease data into broader digital health infrastructures. Continued harmonization and the early adoption of international standards remain key to unlocking the full potential of registry data for patient-centered research and care. This work demonstrates that data harmonization is not just a technical necessity, but a strategic enabler for meaningful research and improved healthcare outcomes in the field of rare diseases. By extending semantic alignment towards automated data transfer, the theoretical alignment demonstrated here represents a first step toward harmonized rare disease data infrastructures and supports the transformation of fragmented rare disease registries into a unified and actionable resource for research.

## Supplementary information

Supplementary Table S1

## Data Availability

The full semantic mapping table generated and/or analysed during the current study are available at https://doi.org/10.5281/zenodo.17712593. The data is provided in a machine-readable format and includes mapping categories as described in the manuscript. The data set from the National Registry for Rare Diseases (NARSE) is available at https://www.narse.de/fileadmin/narse/2025-01-30_NARSE_Datenelemente_DE_V1.2.pdf. The data set is only available in German, but a translation can be found in the Zenodo repository. The modules of the Medical Informatics Initiative core data set can be found on https://github.com/medizininformatik-initiative/kerndatensatz-meta/wiki/Übersicht-über-Versionen-der-Kerndatensatz%E2%80%90Module. Based on the semantic mapping, the potential information loss was analysed as presented in Table 5.

## Code availability

No custom code or scripts were generated or used during this study.
