# Supplementary material for: Strengthening Rare Disease Data Readiness: a semantic approach to cross-registry interoperability
Source: Sci Data. 2026 Sep 30;13:1363. doi: 10.1038/s41597-026-08406-6 (PMC13627711; doi:10.1038/s41597-026-08406-6)
Supplement: Supplementary file 1 [file 41597_2026_8406_MOESM1_ESM.pdf]

source_data_element	source_vocabulary	source_vocabulary_category	target_data_element	target_vocabulary	target_vocabulary_category	skos_matching
Condition.code.coding:orphanet	MII	MII IG Diagnose v2025	Diagnosis Rare Disease(s): ORPHAcode	NARSE	Formal Criteria	exactMatch
Condition.code.coding:icd10-gm	MII	MII IG Diagnose v2025	ICD Diagnosis Rare Disease(s): code	NARSE	Formal Criteria	exactMatch
Condition.code.coding:icd10-gm.version	MII	MII IG Diagnose v2025	ICD Diagnosis Rare Disease(s): Version	NARSE	Formal Criteria	exactMatch
Condition.code.coding:icd10-gm.extension:Diagnosesicherheit	MII	MII IG Diagnose v2025	Diagnostic Diagnosis Rare Disease(s): certainty	NARSE	Formal Criteria	closeMatch
NULL	MII	NULL	Biological sex at birth	NARSE	Personal and family background	no_mapping
Patient.gender	MII	MII IG Person v2025	Administrative Gender	NARSE	Personal and family background	closeMatch
Observation.effectiveDateTime	MII	MII IG Person v2025	Current Status: Date	NARSE	Personal and family background	closeMatch

Patient.deceasedBoolean	MII	MII IG Person v2025	Current status: Status	NARSE	Personal and family background	closeMatch
Patient.deceasedDateTime	MII	MII IG Person v2025	Age at death	NARSE	Personal and family background	closeMatch
Condition.code.coding	MII	MII IG Person v2025	Died of RD	NARSE	Personal and family background	relatedMatch
Condition.code	MII	MII IG Person v2025	Other cause of death	NARSE	Personal and family background	relatedMatch
FamilyMemberHistory.relationship	MII	MII IG MolGen v2025 DE	Affected family members: Relation	NARSE	Personal and family background	closeMatch
FamilyMemberHistory.condition	MII	MII IG MolGen v2025 DE	Affected family members: Rare disease	NARSE	Personal and family background	relatedMatch
FamilyMemberHistory.deceased.deceasedBoolean	MII	MII IG MolGen v2025 DE	Affected family members: Deceased?	NARSE	Personal and family background	exactMatch
Condition.onset[x]:onsetPeriod.start	MII	MII IG Symptom DE v2024	Age at first symptoms	NARSE	Anamnesis and diagnostics	closeMatch
Conditon.onsetPeriod.start	MII	MII IG Symptom DE v2024	Age at first symptoms: Years + Months	NARSE	Anamnesis and diagnostics	closeMatch

Condtion.recordedDate	MII	MII IG Diagnose v2025	Age at diagnosis	NARSE	Anamnesis and diagnostics	closeMatch
Condtion.recordedDate	MII	MII IG Diagnose v2025	Age at diagnosis: Years + Months	NARSE	Anamnesis and diagnostics	closeMatch
Procedure.code.coding | Condition.evidence.code	MII	MII IG Prozedur v2025<br>MII IG Diagnose v2025	Diagnostics	NARSE	Anamnesis and diagnostics	closeMatch
DiagnosticReport.category:Genetics.coding	MII	MII IG MolGen v2025 DE	Genetic diagnosis: Affected gene	NARSE	Anamnesis and diagnostics	closeMatch
LogicalModelMolGen.Ergebnisse.Veraenderungen	MII	MII IG MolGen v2025 DE	Genetic diagnosis: Gene variant	NARSE	Anamnesis and diagnostics	closeMatch
Symptom.Condition.Code | Symptom.Observation.Code	MII	MII IG Symptom DE v2024	Phenotype: HPO term	NARSE	Anamnesis and diagnostics	exactMatch
DiagnosticReport.category.coding:loinc-lab | DiagnosticReport.code.coding	MII	MII IG Laborbefund DE v2025	Laboratory diagnostics: Laboratory parameter	NARSE	Anamnesis and diagnostics	closeMatch

Procedure.code.coding:ops | Procedure.code.coding:sct | Procedure.code.text	MII	MII IG Prozedur v2025	Other diagnostics	NARSE	Anamnesis and diagnostics	closeMatch
Procedure.code.coding:ops | Procedure.code.coding:sct	MII	MII IG Prozedur v2025	Therapy status	NARSE	Anamnesis and diagnostics	closeMatch
Procedure.code.text	MII	MII IG Prozedur v2025	Other specific therapy	NARSE	Anamnesis and diagnostics	closeMatch
Patient.name.given	MII	MII IG Person v2025	First name(s)	NARSE	Identifying data	exactMatch
Patient.name.family.extension.nachname	MII	MII IG Person v2025	Last name(s)	NARSE	Identifying data	exactMatch
Patient.name:geburtsname	MII	MII IG Person v2025	Birth name	NARSE	Identifying data	exactMatch
Patient.birthDate	MII	MII IG Person v2025	Date of birth	NARSE	Identifying data	exactMatch
NULL	MII	NULL	Place of birth	NARSE	Identifying data	no_mapping
Patient.address	MII	MII IG Person v2025	Address	NARSE	Identifying data	exactMatch

Diagnosis Rare Disease(s): ORPHAcode	NARSE	Formal Criteria	Diagnosis of the rare disease: ORPHAcode	ERDRI	Diagnosis	exactMatch
ICD Diagnosis Rare Disease(s): code	NARSE	Formal Criteria	Diagnosis of the rare disease: ICD-10-Code	ERDRI	Diagnosis	exactMatch
Biological sex at birth	NARSE	Formal Personal and family background	Sex	ERDRI	Personal Information	closeMatch
Current status: Status	NARSE	Formal Personal and family background	Patient's status	ERDRI	Patient Status	closeMatch
Age at death	NARSE	Formal Personal and family background	Date of death	ERDRI	Patient Status	closeMatch
Age at first symptoms	NARSE	Anamnesis and diagnostics	Age at onset	ERDRI	Disease history	closeMatch
Age at first symptoms: Years + Months	NARSE	Anamnesis and diagnostics	Age at onset	ERDRI	Disease history	closeMatch
Age at diagnosis	NARSE	Anamnesis and diagnostics	Age at diagnosis	ERDRI	Disease history	closeMatch
Age at diagnosis: Years + Months	NARSE	Anamnesis and diagnostics	Age at diagnosis	ERDRI	Disease history	closeMatch

Genetic diagnosis: Affected gene	NARSE	Anamnesis and diagnostics	Genetic diagnosis	ERDRI	Diagnosis	closeMatch
Genetic diagnosis: Gene variant	NARSE	Anamnesis and diagnostics	Genetic diagnosis	ERDRI	Diagnosis	closeMatch
Phenotype: HPO term	NARSE	Anamnesis and diagnostics	Undiagnosed case	ERDRI	Diagnosis	relatedMatch
Date of birth	NARSE	Identifying data	Date of birth	ERDRI	Personal Information	exactMatch
NULL	NARSE	NULL	Classification of funtioning/disability	ERDRI	Disability	no_mapping
